# Progress and Challenges in Developing Aptamer-Functionalized Targeted Drug Delivery Systems

**DOI:** 10.3390/ijms161023784

**Published:** 2015-10-09

**Authors:** Feng Jiang, Biao Liu, Jun Lu, Fangfei Li, Defang Li, Chao Liang, Lei Dang, Jin Liu, Bing He, Shaikh Atik Badshah, Cheng Lu, Xiaojuan He, Baosheng Guo, Xiao-Bing Zhang, Weihong Tan, Aiping Lu, Ge Zhang

**Affiliations:** 1 Institute for Advancing Translational Medicine in Bone & Joint Diseases, School of Chinese Medicine, Hong Kong Baptist University, Hong Kong, China; E-Mails: jiangfenghz@163.com (F.J.); 13261526031@163.com (B.L.); ljaaa111@163.com (J.Lu); fayebalaba@live.com (F.L.); lidefang@163.com (D.L.); liangchao512@163.com (C.Liang); danglei_hkbu@163.com (L.D.); liujin_hkbu@163.com (J.Liu); hebinghb@gmail.com (B.H.); aatikshaikh@gmail.com (S.A.B.); lv_cheng0816@163.com (C.Lu); hxj19@126.com (X.H.); boris.g.guo@gmail.com (B.G.); 2 Hong Kong Baptist University Branch of State Key Laboratory of Chemo/Biosensing and Chemometrics of Hunan University, Hong Kong, China; 3 Molecular Science and Biomedicine Laboratory, State Key Laboratory of Chemo/Bio-Sensing and Chemometrics, College of Chemistry and Chemical Engineering, Hunan University, Changsha 410000, China; 4 College of Biology, Hunan University, Changsha 410000, China

**Keywords:** aptamer-functionalized targeted drug delivery systems, aptamer, SELEX, aptamer-small molecule conjugated systems, aptamer-nanomaterial conjugated systems

## Abstract

Aptamers, which can be screened via systematic evolution of ligands by exponential enrichment (SELEX), are superior ligands for molecular recognition due to their high selectivity and affinity. The interest in the use of aptamers as ligands for targeted drug delivery has been increasing due to their unique advantages. Based on their different compositions and preparation methods, aptamer-functionalized targeted drug delivery systems can be divided into two main categories: aptamer-small molecule conjugated systems and aptamer-nanomaterial conjugated systems. In this review, we not only summarize recent progress in aptamer selection and the application of aptamers in these targeted drug delivery systems but also discuss the advantages, challenges and new perspectives associated with these delivery systems.

## 1. Introduction

Many drugs that are used in clinical treatment, particularly anticancer drugs, do not explicitly discriminate between pathological cells and normal cells. The indiscriminate exposure of normal cells to these drugs often leads to the use of a suboptimal dosage and is responsible for many of the toxicities associated with these drugs. Combining this issue with the emergence of drug-resistant pathological cells, it is clear that selective and efficacious therapies with minimal toxicity are urgently required [1]. It is known that targeted drug delivery systems can improve the specificity of delivered agents for an intended location, can increase the accumulation in the cellular of disease tissue and can potentially provide an alternative delivery mechanism [2]. Therefore, such systems are desirable and have become an exciting area of research in recent decades, and hundreds of approaches and strategies for selectively delivering potent drugs to pathological cells have been developed. Targeted drug delivery systems are especially of significance for drug delivery to tumors, as evidenced by the fact that certain drugs have now been approved for oncological indications by the Food and Drug Administration (FDA) [1,3].

Targeted drug delivery systems are simple but efficient and are composed of therapeutic agents and ligands that undergo noncovalent or covalent conjugation for targeted delivery. To achieve effective drug delivery, the first step is to develop a suitable ligand that can recognize the morphological and physiological differences between pathological and normal tissues. Early work in targeted drug delivery systems used polyunsaturated fatty acids, folic acid, hyaluronic acid or oligopeptides as tumor recognition moieties. However, research on these ligands is at a standstill for a variety of reasons, such as the need to clarify the detailed mechanism of tumor targeting by polyunsaturated fatty acids; the fact that the application of folic acid in small-size conjugates has only limited success [4]; and the observation that peptides are susceptible to enzymatic degradation in the systemic circulation, making them unsuitable for many *in vivo* applications [5].

Nucleic acid aptamers are identified through an *in vitro* selection process called systematic evolution of ligands by exponential enrichment (SELEX) [6]. Since their discovery in the 1980s, aptamers have attracted considerable interest for medical applications as therapeutic agents, diagnostic tools and moieties for targeted drug delivery [7]. In particular, aptamers are short, single-stranded DNA (ssDNA) or RNA oligonucleotides with specific secondary and tertiary structures, which exert their biological and physiological effects by binding to targeted proteins with high affinity and specificity [8]. Due to their specificity, low immunogenicity and toxicity, easily modified chemical structure and wide range of targets, aptamers are superior ligands encouraging the development of aptamer-targeted drug delivery systems.

Depending on their different compositions and preparation methods, aptamer-targeted drug delivery systems can be divided into two main categories: aptamer-small molecule conjugated systems (in which aptamers directly deliver drug molecules as both a carrier and a ligand) and aptamer-nanomaterial conjugated systems (in which aptamers function together with nanoparticles (NPs) for targeted delivery of drugs) [9]. This review is focused on the recent advances in the development of aptamer SELEX, aptamer-small molecule conjugated systems and aptamer-nanomaterial conjugated systems.

## 2. Aptamer SELEX

SELEX is a well-established and efficient technology for the screening of oligonucleotides with high affinities for their targets from random-sequence libraries [10]. This technique was introduced in 1990 by Andrew Ellington and Larry Gold and has been an important tool ever since for the identification and screening of aptamers. In fact, a wide variety of aptamers have been identified using the SELEX technique since the first report on SELEX 20 years ago [11]. After decades of development, this method has undergone dramatic changes and improvements. In addition to conventional SELEX [12,13,14], there are improved versions such as capillary electrophoresis-SELEX [15,16,17], magnetic bead-based SELEX [18,19,20], cell-SELEX [21,22,23,24,25,26,27], automated SELEX [28,29,30,31], complex-target SELEX [32,33,34,35], and so on. Table 1 shows some examples of nucleic acid aptamers that bind to targets of therapeutic interest. Since there are already many published reviews on aptamer SELEX [12,24,29,36], in this section, we highlight the cell-SELEX and complex-target SELEX strategy, which select aptamers able to bind to a specific cell type or a complex-target.

**Table 1:** Example of nucleic acid aptamers.

Aptamer	Molecular Target	Associated Disease	Aptamer Structure	Ref.
Macugen	Vascular endothelial growth factor (VEGF)	Age-related macular degeneration	5′-CGGAAUCAGUGAAUG CUUAUACAUCCG-3′	[37]
AS1411	Nucleoin	Cancer	5′-d(GGTGGTGGTGGT TGTGGTGGTGGTGG)-3′	[38]
Sgc8	Protein tyrosine kinase 7 (PTK-7)	Cancer	5′-ATCTAACTGCTGC GCCGCCGGGAAAATA CTGTACGGTTAGA-3′	[39]
TD05	Immunoglobulin μ heavy chains (IGHM)	Lymphoma	5′-AACACCGGGAGGAT AGTTCGGTGGCTGTTCA GGGTCTCCTCCCGGTG-3′	[40]
ARC1779	A1 Domain of von Willebrand factor (vWF)	Thrombotic microangiopathies and carotid artery disease	5′-GCGUGCAGUGCCU UCGGCCGTGCGGT GCCUCCGUCACGC-3′	[41]
TBA	α-Thrombin	Thrombosis	5′-GGTTGGTGTGGTTGG-3′	[42]

### 2.1. Cell-SELEX

**Figure 1:**
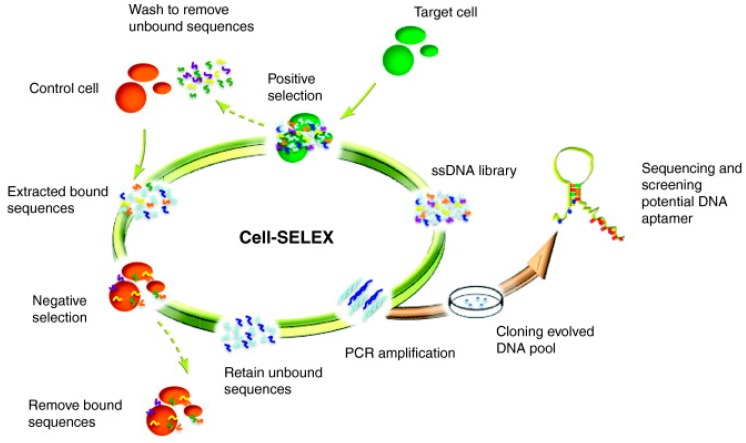
Schematic illustration of the cell-SELEX (systematic evolution of ligands by exponential enrichment) process. Reproduced with permission from Reference [43].

In this method, to identify a cell-specific aptamer, cells of a certain type can be used as positive targets, and normal cells can be used as negative targets [21]. The screening process of Cell-SELEX is as follows. First, an oligonucleotide library with random sequences is constructed with constant primers flanking the 5′ and 3′ ends [22]. The total size of the library can be as large as 10^14^, covering nearly all of the possible three-dimensional conformations that can be applied to target nearly all types of natural molecules [23]. The oligonucleotide library is then incubated with target cells at a certain temperature, and the aptamers that bind to target cells are isolated as a library for negative selection. Meanwhile, the aptamers that bind to both target cells and non-target cells are removed. Finally, the aptamers are washed and amplified by PCR or RT-PCR to generate a secondary library for the next round of screening [24,25]. The three major steps of cell-SELEX, including incubation, partitioning and amplification, are shown in Figure 1. Using this method, Lu and Zhang’s group [26] specifically selected aptamers from a library composed of 10^15^ different ssDNA sequences. In this study, rat primary osteoblasts were used as target cells, whereas a rat liver cell line BRL-3A and rat PBMCs were used as negative targets. The researchers obtained an aptamer that specifically recognized and bound to the target cells after only 14 rounds of selection, and they successfully applied the aptamer to the development of an osteoblast-specific delivery system.

Compared with conventional SELEX technology, Cell-SELEX exhibits the following advantages. First, through this process, functional aptamers against various targets—ranging from small targets such as proteins and peptides to complex targets such as complete tumor cells, bacteria and other pathogens—can be isolated [27]. Second, this well-developed method uses whole cells as targets. Based on this principle, it is feasible to screen aptamers specifically recognizing target cells as long as there are different expression levels of target molecules between the positive and negative target cells, even without information about the specific target molecule. Third, when using whole cells as targets, the target protein on the cell surface can retain its native conformation. Aptamer selection in this context can facilitate the identification of biomarkers expressed on target cells.

### 2.2. Complex-Target SELEX

Early targets for SELEX were usually purified soluble proteins. However, it is difficult to screen aptamers with only one protein as the target, as a protein normally functions by forming a complex with other proteins, which is the case for transmembrane proteins or ion channels [32]. Thus, it was necessary to develop a novel approach to screen aptamers against complex targets. In particular, complex-target SELEX can be used to screen different aptamers against multiple molecules in parallel [33]. However, if the target protein is just one part of a composite target, other methods, such as subtractive SELEX, should be applied to eliminate interference factors. Morris’s group used complex-target SELEX to identify an ssDNA aptamer that could bind to red blood cells after 25 rounds of selection, and they demonstrated that different aptamers could specifically recognize different molecular targets [34]. Similar results were reported for aptamer screening against human plasma and γ-carboxyglutamic acid protein complex targets. Shamah [35] summarized recent studies performing aptamer screening using complex-target SELEX and also discussed the process for selecting aptamers against composite targets, including cell debris, cell membranes, bacteria, parasites, viruses and animal cells. This approach can also be applied in a selection process using a membrane protein as the target: the membrane protein is expressed on the cell surface through genetic engineering, and complex-target SELEX can then be performed for aptamer selection.

The development of complex-target SELEX greatly enriched the pool of aptamer screening targets and expanded the potential applications of aptamers in disease diagnosis, drug development and other research fields.

Although aptamer is an excellent candidate for targeted drug delivery, the wild-type RNA and DNA molecules can be easily degraded by nucleases [44]. In order to increase the stability of the aptamer, several schemes have been proposed, such as modification of aptamer with 2′-aminopyrimidine [45], 2′-fluoropyrimidine [45], or 2′-*O*-methyl nucleotides [46]. Precise site-specific modifications facilitate the engineering of aptamers for improved specificity and stability. The mechanism of action for aptamers and the reasons for their marked tumor selectivity were also studied. Bates *et al.* [47,48,49,50] found that AS1411 is a quadruplex forming oligodeoxynucleotide that binds to nucleolin. The nucleolin is not only serving as the receptor for AS1411 and leading to selective uptake in cancer cells, but also essential for the stimulation of macropinocytosis of AS1411 which leads to further uptake of the aptamer. In cancer cells, uptake by macropinocytosis allows endosomal escape of AS1411, and AS1411 induces cell death due to hyperstimulation of macropinocytosis.

In summary, after years of improvement and development, aptamer-screening technology has achieved fruitful progress in many respects. Many new SELEX methods have been developed for an easier and faster selection of aptamers, and both the screening efficiency and the range of application have been improved. However, each method has its limitations, including a long screening time, high labor requirements and/or high cost. Additionally, there is still a lack of a common screening method. Therefore, it is necessary to develop new technologies to solve these problems. In the future, the core work of aptamer screening should focus on the following aspects: first, the mechanisms by which aptamers have affinity for their target molecules; second, new methods to shorten the screening cycle and to reduce screening costs; and third, the establishment of a more optimal aptamer-screening platform to improve the degree of automation.

## 3. Aptamer-Small Molecule Conjugated Systems

Because targeted drugs that undergo nonchemical conjugation are unstable due to the reversible nature of the noncovalent conjugation process [36], aptamer-small molecule conjugated systems mainly refer to aptamer-drug conjugates with covalent linkers. Currently, aptamer-small molecule conjugates are still in the early stages of development. One well-known example is sgc8c-doxorubicin (DOX), developed by Tan and colleagues [51]. Sgc8c is a DNA aptamer that specifically binds to protein tyrosine kinase 7 (PTK7) on the surface of CCRF-CEM (T-cell acute lymphoblastic leukemia, T-cell ALL) cells with high binding affinity. The study showed that sgc8c-DOX conjugate possess high binding affinity of the sgc8c aptamer, and could be efficiently internalized by target cells. The sgc8c-DOX conjugate not only demonstrates anticancer potency similar to unconjugated DOX, but also exhibits limited toxicity toward non-target cells. Another aptamer-small molecule conjugate was also developed by Tan and colleagues, who designed and synthesized a therapeutic module via solid-phase synthesis, consisting of a phosphoramidite containing an anticancer drug moiety and a photocleavable linker [52]. Such examples demonstrated that aptamer-small molecule conjugates could achieve targeted drug delivery similar to antibody-drug conjugates (ADCs). An aptamer-small molecule conjugate consists of three main components—an aptamer, a linker and a small molecule drug (also frequently referred as the payload)—and its development relies on two key research areas: the selection of highly potent but cytotoxic drugs and the application of appropriate linkers. In our opinion, the drugs and linkers applied in ADCs may also be suitable for aptamer-drug chemical conjugates. Therefore, in this section, we review recent research advances in these two areas related to ADCs and ApDCs.

### 3.1. Cytotoxic Drugs

The selection of cytotoxic drug is critical because it affects efficacy toward the targeted tumor. Early research focused on well-established and clinically approved cytotoxic drugs, such as DOX, methotrexate, mitomycin, 5-fluorouracil, and Vinca alkaloids. However, the low efficacies of such ADCs in preclinical and clinical evaluations showed that these drugs had limited potency, which encouraged the use of much more potent drugs that were too toxic to be used in an untargeted manner in ADCs [53]. Today, two main categories of highly potent cytotoxic anticancer agents are used in ADCs: DNA-modifying agents and microtubule-disrupting agents, which demonstrate at least 100–1000-fold greater potency compared with conventional chemotherapeutic agents [54].

**Figure 2:**
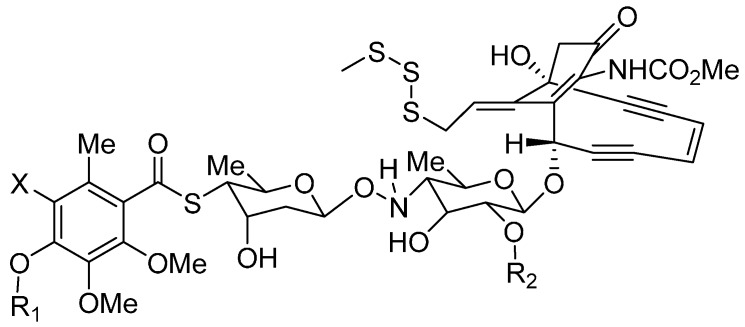
The structural formula of calicheamicins. Adapted from Reference [55].

**Figure 3:**
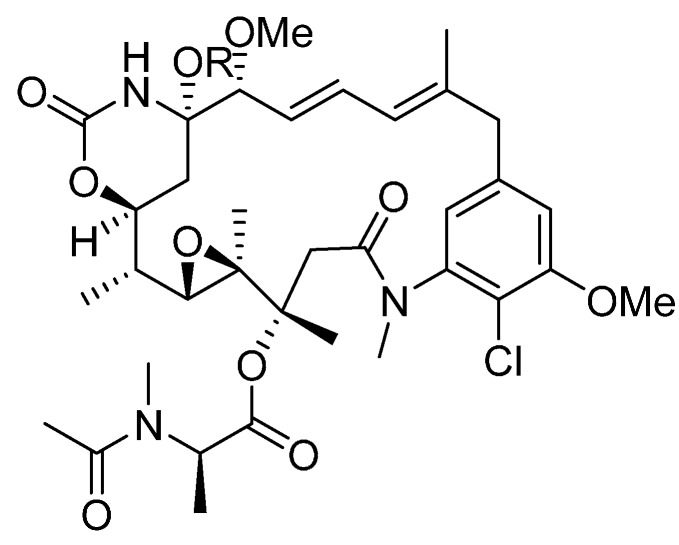
The structural formula of maytansines. Adapted from Reference [56].

**Figure 4:**
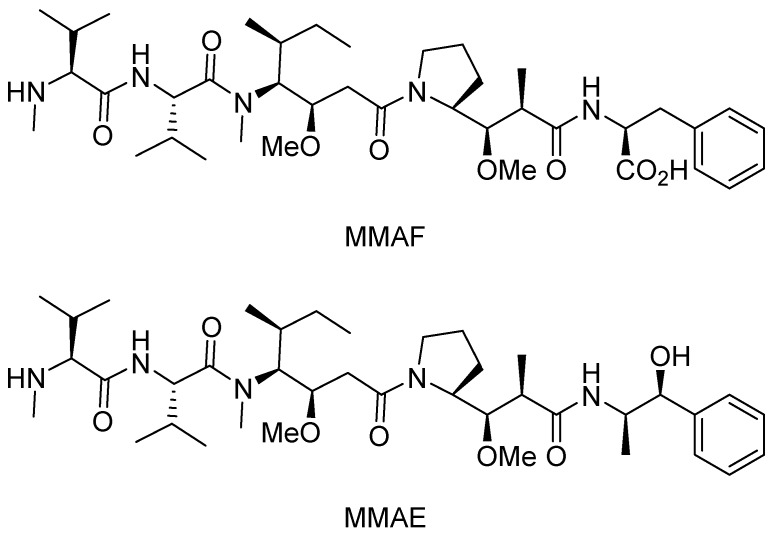
The structural formulas of monomethyl auristatin F (MMAF) and monomethyl auristatin E (MMAE). Adapted from Reference [57].

The calicheamicins, which were originally isolated from a broth extract of the soil microorganism *Micromonospora echinospora* ssp. calichensis, are DNA-modifying agents that represent a novel structural class with powerful biological properties that confer potent antitumor activity (Figure 2). For example, calichensis shows extraordinary potency against murine tumors and is approximately 4000-fold more active than adriamycin, with an optimal dose of 0.5–1.5 μg/kg [55]. Maytansine, isolated from *Maytenus* species, is a microtubule-disrupting payload that inhibits the microtubule assembly, induces the microtubule disassembly and disrupts mitosis (Figure 3) [56]. Additionally, dolastatin 10 is a microtubule-disrupting agent for cancer chemotherapy that was isolated from the sea hare *Dolabella auricularia* [57]. Since these agents were first studied, many research groups have engaged in structure activity relation (SAR) studies of their synthetic analogs, termed “auristatins”, such as monomethyl auristatin F (MMAF) and monomethyl auristatin E (MMAE) (Figure 4). The auristatins exhibit picomolar GI_50_ values in most cancer cell proliferation assays, caused by their ability to strongly inhibit microtubule assembly and tubulin-dependent GTP hydrolysis, resulting in cell cycle arrest and apoptosis [58].

More recently, two promising cytotoxic agents were investigated: pyrrolobenzodiazepines (PBDs), which form dimers that bind to the minor groove of DNA in a sequence-selective manner [59,60], and the RNA polymerase II inhibitor α-amanitin [61]. Cytotoxic agents used in aptamer-drug chemical conjugates should possess the following properties: (1) high *in vitro* potency toward tumor cell lines, with IC_50_ values within the range of 0.01–0.1 nM; (2) a suitable functional group for linkage to a ligand (if a functional group is not already present, the desired substituent must be introduced at a suitable site to retain the potency of the parent drug); (3) reasonable solubility in aqueous solutions to enable reaction with the ligand; and (4) prolonged stability in the aqueous formulations commonly used for the ligand [62].

### 3.2. Linkers

One of the biggest challenges in the development of chemically conjugated targeted drugs is the application of suitable linkers for conjugating a ligand and a drug [63]. With the development of ADCs, a series of linkers had already been exploited. The linker that connects a cytotoxic drug to a ligand should be relatively stable in circulation to limit the damage caused to healthy tissues by highly active agents before they are delivered to the target site. Once the chemically conjugated targeted drug is internalized, however, the linker should facilitate efficient drug release. Based on the chemical structure and properties of the conjugated drug, three types of linkers can be selected: chemically labile linkers, enzyme-labile linkers and non-cleavable linkers.

#### 3.2.1. Chemically Labile Linkers

##### Acid-Cleavable Linkers

Acid-cleavable linkers were the first type to be used. These linkers take advantage of the low pH in endosomes and lysosomal compartment to trigger hydrolysis of an acid-cleavable portion, such as a hydrazone bond, to release the payload.

**Figure 5:**
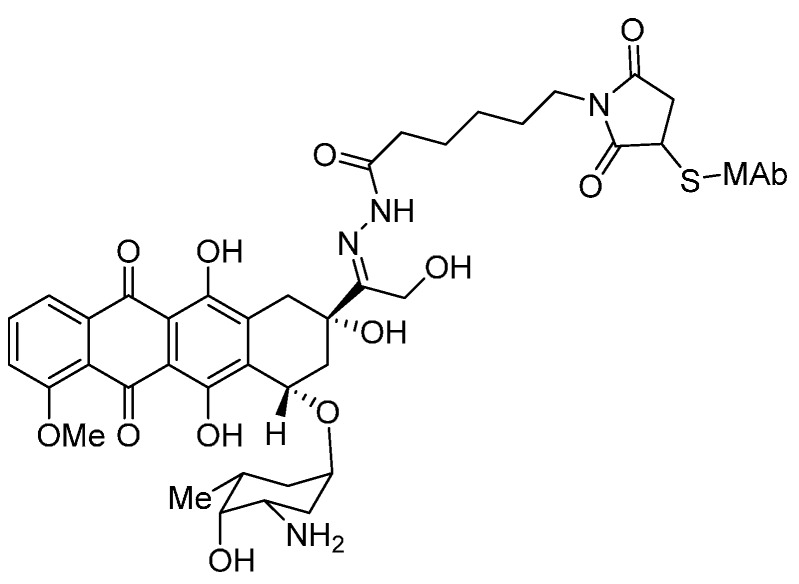
The structural formula of BR96-doxorubicin. Adapted from Reference [64].

A prominent example of an acid-cleavable linker-based ADC is BR96-DOX (Figure 5), which has curative efficacy in human tumor xenograft models. However, in clinical trials, significant toxicity was observed due to nonspecific cleavage of the relatively labile hydrazone linker and the expression of the antigen target in normal tissue [64]. The development of DOX-based ADCs may still continue, and milatuzumab-DOX with a hydrazone bond linker, is currently in the advanced stages of clinical trials.

Mylotarg was the first ADC ever approved by the FDA and is used as a second-line therapy for the treatment of relapsed acute myelocytic leukemia. This ADC consists of a humanized anti-CD33 monoclonal antibody (MAb) (hP67.6) attached to a calicheamicin drug through an acid-labile hydrazone linker. However, this linker is relatively unstable in plasma, with a half-life of approximately 48–72 h [65]. A hydrazone linker was also used in inotuzumab ozogamicin (an anti-CD22 calicheamicin conjugate). Despite using the same linker and payload as Mylotarg, this ADC appears to be more stable. This conjugate is also currently undergoing clinical evaluation [66].

The linker was also applied in ApDC to conjugate the aptamer Sgc8c and DOX, the resulting conjugate showed similar anticancer potency to unconjugated DOX [51].

##### Disulfide Linkers

The use of this type of linker is attractive because the concentration of glutathione in tumor cells is much higher than that in normal cells. In particular, irregular blood flow results in both a hypoxic environment in the tumor and enhanced activity of reductive enzymes, therefore leading to higher glutathione concentrations [67]. Thus, the linker, which is stable in circulation, can efficiently reduce the release of the active drug from the non-toxic prodrug at the tumor site, and elevate the stability by increasing steric hindrance [68].

**Figure 6:**
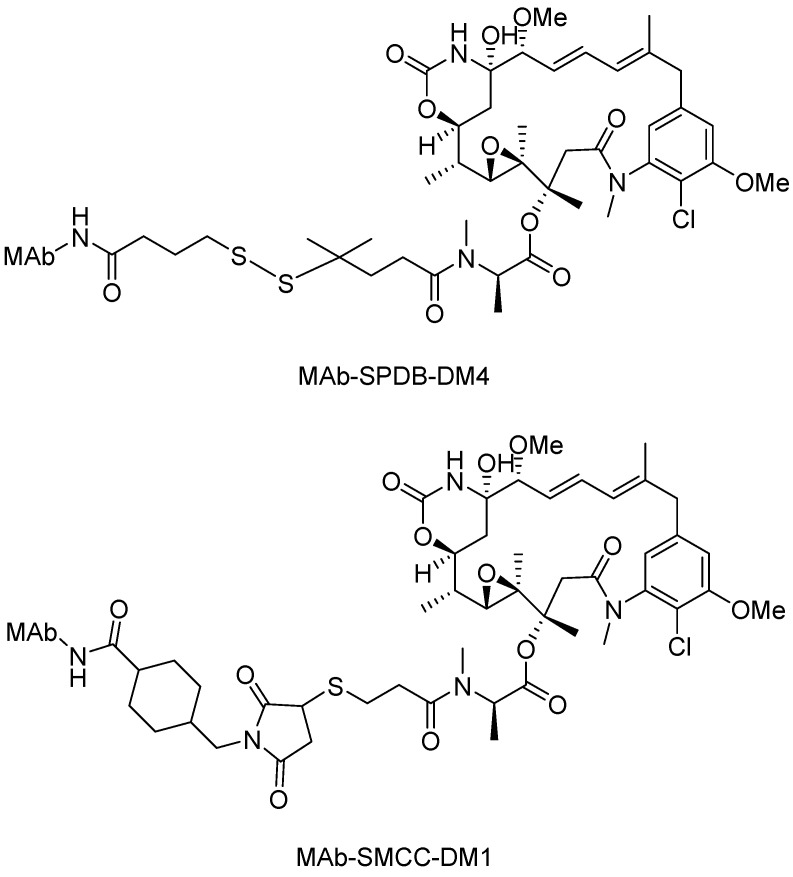
The structural formulas of huC242-SPDB-DM4 and huC242-SMCC-DM1. Adapted from Reference [70].

Representative disulfide linker-based conjugates include maytansine derivatives conjugated to different MAbs. One maytansine derivative, DM1, has been conjugated to trastuzumab via a disulfide bond, allowing targeted delivery to tumor cells overexpressing human epidermal growth factor receptor 2 (HER2) [69]. The comparison between these antibody-maytansinoid conjugates with a “cleavable” linker containing a disulfide bond and a “non-cleavable” linker containing a thioether were shown in Figure 6. The conjugate huC242-SMCC-DM1, with a non-cleavable linker, was at least as potent *in vitro* as the conjugate huC242-SPDB-DM4, with a disulfide bond. However, huC242-SMCC-DM1 exhibited significantly lower *in vivo* activity in multiple xenograft tumor models [70,71]. The conjugates of huC242 with DM1 which linked via a disulfide bond could exert the bystander effect, whereas the conjugates with thioether bond could not [72]. Moreover, the clearance rate of the conjugate containing a reducible disulfide bond was approximately four times faster than that of the antibody component. The covalent linkage of 3–4 maytansinoid drug molecules to huC242 did not change the distribution of the antibody in mice [73].

Certain representative maytansine ADCs containing a reducible disulfide bond as a linker are currently undergoing clinical evaluation: SAR3419, lorvotuzumab mertansine (IMGN901), BT-062, BAY-94-9343 and SAR-566658.

#### 3.2.2. Enzyme-Labile Linkers

##### Peptide Linkers

Peptide linkers, as the name suggests, attach a drug to an antibody via a peptide bond. Because proteases are normally not active outside cells due to unfavorable pH conditions and serum protease inhibitors, these peptide linkers are expected to have good serum stability. However, these linkers can be cleaved by lysosomal proteases, leading to elevated levels of free drug in certain tumor tissues. Many ADCs designed to utilize this linker strategy thus display an optimal balance between plasma stability and intracellular protease cleavage.

**Figure 7:**
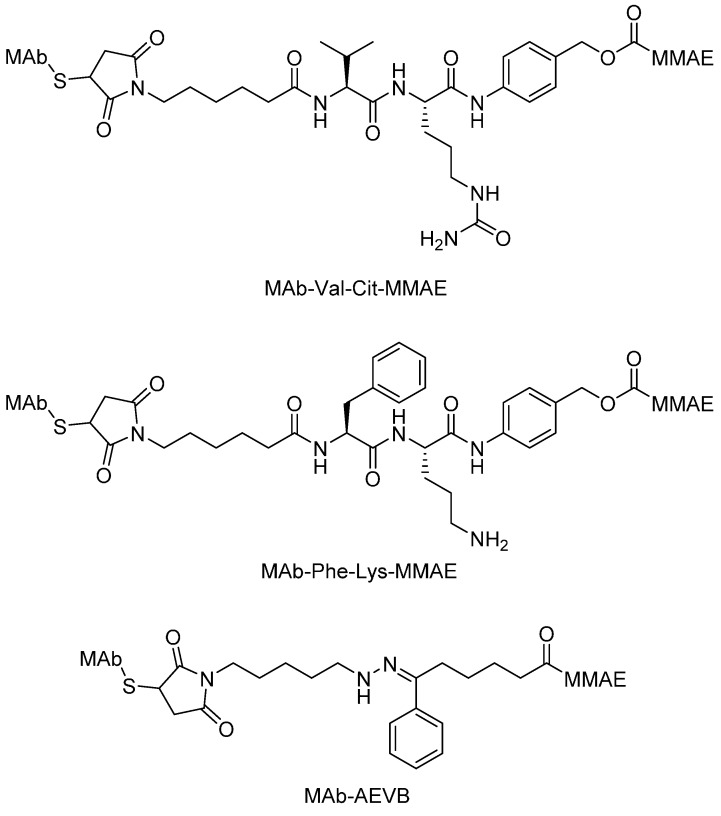
The structural formulas of MAb-Val-Cit-MMAE, MAb-Phe-Lys-MMAE and MAb-AVEB. Adapted from Reference [74,75,76].

**Scheme 1:**
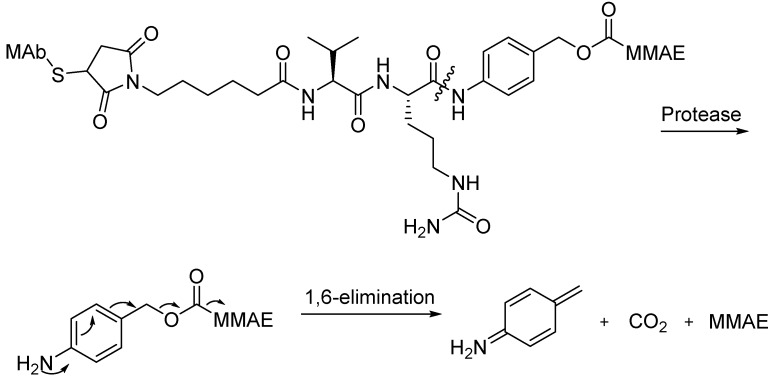
Presumed release mechanism of MMAE from an ADC incorporating a peptide linker. Adapted from Reference [74,75].

The most well-known conjugates involving a peptide linker are the MMAE and MMAF ADCs. Wahl described the *in vitro* and *in vivo* properties of MAb-drug conjugates consisting of auristatin E (AE) and MMAE linked to the chimeric MAbs cBR96 and cAC10 via an acid-labile hydrazone and protease-sensitive dipeptides. The researchers found that the peptide-linked MAb-valine-citrulline (Val-Cit)-MMAE and MAb-phenylalanine-lysine(Phe-Lys)-MMAE conjugates were much more stable in buffers and plasma compared with the MAb conjugates linked via the hydrazone of 5-benzoylvaleric acid-AE ester (AEVB) (Figure 7). The MAb-Val-Cit-MMAE conjugates released only 2% of MMAE in the following 10 days after being incubated in human plasma, whereas they could easily be cleaved by lysosomal protease after internalization (Scheme 1). Compared with the corresponding hydrazone conjugates, the MAb-Val-Cit-MMAE conjugates showed greater *in vitro* specificity and lower *in vivo* toxicity. The therapeutic indices were shown to be as high as 60-fold for the peptide-linked conjugates induced regression and cure of established tumor xenografts. The treatment with cAC10-Val-Cit-MMAE at 1 mg mAb component/kg/injection and at 0.5 mg/kg/injection resulted in 100% and 80% tumor inhibition, respectively [74,75,76].

In July 2011, Adcetris, which consists of an anti-CD30 MAb attached through a Val-Cit dipeptide to an average of four MMAE molecules, was approved by the FDA for the treatment of patients with Hodgkin’s lymphoma after failed autologous stem cell transplantation, or patients with systemic anaplastic large-cell lymphoma after the failure of at least one prior multi-agent chemotherapy regimen [65]. There are also certain representative MMAE- or MMAF-containing ADCs containing a peptide bond linker that are undergoing clinical evaluation now: RG-7596, glembatumumab vedotin (CDX-011), PSMA-ADC and ASG-5ME.

##### β-Glucuronide Linkers

This type of linker is exploited such that drug release requires cleavage by β-glucuronidase, an enzyme present in lysosomes and the tumor interstitium [77]. The linker is hydrophilic, so it could reduce the ADC aggregation of the hydrophobic drugs [78].

Cyclopropyl indole minor-groove binders (MGBs) have been conjugated to the MAbs cAC10 (anti-CD30) and h1F6 (anti-CD70) using a β-glucuronide linker. The resulting ADCs were non-aggregated and monomeric, even when heavily loaded with hydrophobic anticancer drugs (up to eight drugs/MAb). The free drug was released through the cleavage of the β-glucuronide linker [79].

This linker was also used in ADCs bearing camptothecin analogs, and the ADCs showed high potency, immunologically specificity and efficacy at well-tolerated doses in a renal cell carcinoma xenograft model [80].

#### 3.2.3. Non-Cleavable Linkers

Non-cleavable linkers have also proven to be very potent. The release mechanism is believed to occur via internalization of the ADC, followed by the degradation in the lysosome, resulting in the release of the drug while still attached to the linker. As such, when the payloads could maintain the antitumor effects even if being chemically modified, the non-cleavable linker is a good choice.

**Figure 8:**
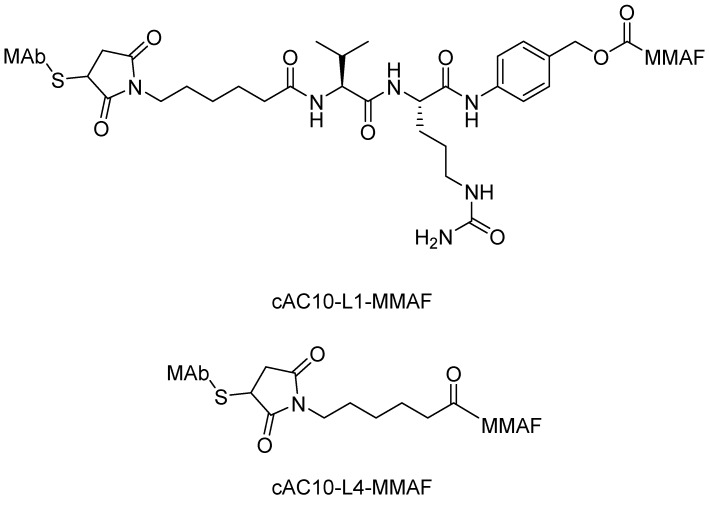
The structural formulas of cAC10-L1-MMAF and cAC10-L4-MMAF. Adapted from Reference [81,82].

Early studies that described non-cleavable linkers were aimed at amine-to-thiol coupling. One advantage of these linkers is their greater stability in the circulation compared with cleavable linkers, which can potentially improve the therapeutic index because it may be better tolerated. Senter found that MMAF, conjugated to a MAb via a non-cleavable maleimidocaproyl linker (cAC10-L4-MMAF), was approximately as potent as when conjugated via a cleavable peptidic linker (cAC10-L1-MMAF) against a large panel of cell lines both *in vitro* and *in vivo*. Importantly, cAC10-L4-MMAF was tolerated at a dose more than three times higher than the MTD of cAC10-L1-MMAF. The half-lives of such non-cleavable linkers in circulation are much longer than those of earlier-generation cleavable linkers (Figure 8). Nearly all animals that received single ADC injections at 2 mg antibody component/kg body weight were cured. When lowering the dose to 1 mg/kg, there were two of six and three of six animals cured by cAC10-L1-MMAF4 and cAC10-L4-MMAF4 ADCs, respectively [81,82].

When examining the intracellular release of a series of disulfide-linked ADCs, it was unexpectedly found that a trastuzumab-DM1 control conjugate, in which the disulfide was replaced with a non-cleavable thioether bond, was the most effective ADC variant in *in vivo* therapy tests [83].

This type of linker is successfully used in Kadcyla, which was approved by FDA in 2013 and has been used to treat patients with HER2-positive metastatic breast cancer. In addition, IMGN529 containing a thioether bond linkage has entered phase I clinical trials. However, through a retro-Michael reaction, the thioether succinimide can revert to the starting materials thiol and maleimide. The reversibility of this connection between antibody and linker has been demonstrated in several ADCs and has been shown to result in a decrease in the efficacy of the corresponding drug conjugate [80]. In order to solve the problem, there were a series of recent attempts to design more stable linkers [84,85,86,87].

Recently, Zu *et al.* [88] identified a single-strand DNA aptamer specifically binding to the biomarker CD117, which is highly expressed on AML cells, then conjugated the aptamer to methotrexate (MTX) using a non-cleavable bond. The formed Apt-MTX conjugates specifically inhibited AML cell growth and had little effect on CD117-negative cells under the same treatment conditions.

In summary, a series of studies have demonstrated that the appearance of ADCs and ApDCs boosted the development of chemically conjugated targeted drugs. Regarding ADCs in particular, the development of aptamer-drug chemical conjugates has relied on two key areas of research: the choice of potent cytotoxic drugs and the application of appropriate linkers. The drugs selected for a chemical conjugation and targeting should have two characteristics in common: high potency and ease of modification. The exact linker between a cytotoxic agent and a ligand has profound effects on the selectivity, PK, therapeutic index, and overall success of a chemically conjugated targeted drug. Each linker has its advantages and disadvantages, and many factors must be evaluated when selecting an appropriate linker, including the existing groups presented in the carrier and the introduction of special reactive groups into agents. Certain cytotoxic payloads that are not active with substitutions require a cleavable linker. In contrast, other cytotoxic payloads that can accommodate substitutions while maintaining potency are good substrates for the development of non-cleavable linkers.

Although chemically conjugated targeted drugs exhibit certain advantages in terms of the targeted delivery of drugs to tumor cells, certain challenges remain, including low drug loading and costly multistep procedures involved in the design and synthesis. Future work will be necessary to increase drug loading, to reduce costs or to identify a better approach for achieving targeted drug delivery.

## 4. Aptamer-Nanomaterial Conjugated Systems

Nanomaterials have unique physicochemical properties, such as an ultra-small size, a large surface-area-to-mass ratio, and high reactivity, in contrast to bulk materials of the same composition [89]. These properties could be used to overcome several of the limitations of traditional therapeutic and diagnostic agents. These particles allow exquisite modification for binding to target cell membranes, components of the microenvironment, or cytoplasmic/nuclear receptor sites [89]. Combining the advantages of nanomaterials with the high affinity and specificity of aptamers, aptamer-functionalized nanomaterials have drawn great attention in drug delivery. Based on their different compositions, the nanomaterials applied in aptamer-targeted drug delivery systems can be divided into two categories: inorganic and organic nanomaterials. In this section, several representative aptamer-nanomaterial conjugated systems are discussed.

### 4.1. Inorganic Nanomaterials

#### 4.1.1. Gold Nanoparticles

Gold NPs (AuNPs) have attracted considerable attention as drug delivery platforms because of their numerous advantageous properties. First, AuNPs have been shown to be essentially inert, non-toxic, and biocompatible. Second, AuNPs can be easily modified with aptamers due to their predictable and reliable surface modification chemistry, and the resulting AuNPs possess the affinity and specificity of the aptamer. Third, the optical and electronic properties of AuNPs are highly shape- and size-dependent, which has led to their use in many biomedical applications [90,91].

**Scheme 2:**
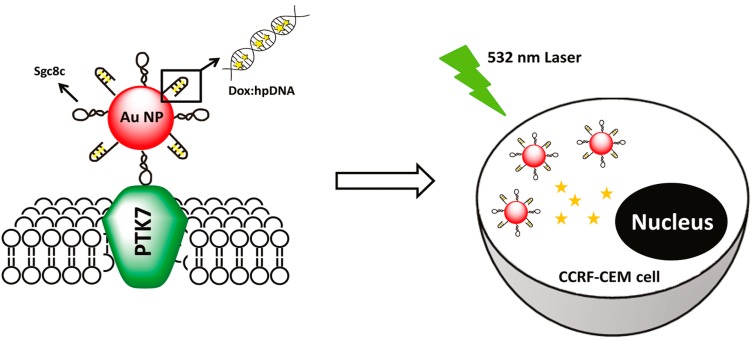
Light-induced Dox release from Dox:apt/hp-Au NP nanocomplexes inside targeted cancer cells (schematic). Reproduced with permission from Reference [92].

Huang *et al.* [92] devised an aptamer/hairpin DNA-AuNP (apt/hp-AuNP) conjugate for targeted drug delivery. The DNA aptamer sgc8c which possesses strong affinity for protein tyrosine kinase 7 (PTK7) was used as the ligand, and the hairpin DNA was used for loading of the anticancer drug DOX. The capability of the apt/hp-AuNP conjugate binding to target cells was investigated by flow cytometry and atomic absorption spectroscopy, which showed that the aptamer-functionalized nanoconjugates were selective for cancer cells. When illuminated with a continuous-wave laser, corresponding to the resonant wavelength of Au NPs, the conjugate could release DOX and more effectively kill targeted cancer cells. Most interestingly, the nanoconjugates could enhance the antitumor efficacy with few side effects when illuminated with plasmon-resonant light (532 nm) (Scheme 2). The inhibition concentration (IC_50_) of Dox:sgc8c/hp AuNPs was quite similar to that of free Dox.

Moreover, Qu *et al.* [93] constructed novel gold nanorods (GNRs) with a mesoporous silica framework (AuMPs). The surfaces of these nanorods were then modified with the AS1411 aptamer (Ap-AuMPs). One oligonucleotide containing the AS1411 aptamer with a 12-base extension was chosen as the capping agent (DNA-2). Another 12-mer oligonucleotide (DNA-1) was covalently attached to the exterior of the mesoporous particles. Thus, the DNA could form an assembly with two identical duplex regions connected by a dimeric G-quadruplex. The duplex portion could serve as a linker anchored to the mesoporous silica NP (MSN) surface, and the G-quadruplex was utilized as a cap for trapping the guest molecules. When exposed to a laser beam matching the absorption peak of the GNRs, the duplex DNA denatured at an increased temperature, allowing the release of the entrapped drug.

**Scheme 3:**
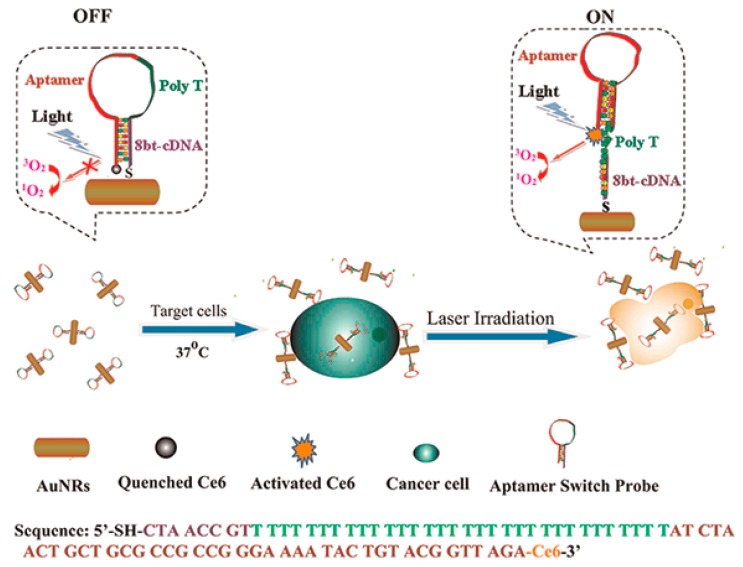
Schematic representation of ASP_photosensitizer_AuNRs for PTT and PDT. Reproduced with permission from Reference [94].

Aptamer-photosensitizer conjugates have been used for photodynamic therapy (PDT), with a high degree of selectivity [94,95]. In particular, Tan *et al.* [94] used the photosensitizer molecule chlorin e6 (Ce6) conjugated to aptamer-GNRs for photothermal therapy (PTT). In this case, aptamer sgc8 which binds the cell membrane protein tyrosine kinase-7 (PTK7) with high affinity and selectivity was used as the ligand. Due to the high expression on cell surface and internalization function of PTK7 in cancer cells, sgc8 was taken up by cells and delivered into the endosomes. When the targeted cancer cells were absent, Ce6 was quenched and demonstrated no PDT effect. However, when the targeted cancer cells were present, the aptamer changed its structure to release Ce6 and produced singlet oxygen for PDT upon light irradiation. PTT/PDT dual therapy presented a more effective therapeutic outcome than either therapeutic modality alone by combining photosensitizer and photothermal agents (Scheme 3). In another case, aptamers were conjugated to the surfaces of GNRs through thiol-gold covalent linkages, and the resulting conjugates were successfully used to target and kill both cancer cells and cancer stem cells by near-infrared (NIR) laser irradiation [95].

However, *in vivo* experiments were not performed in none of the studies. Furthermore, the accumulation and potential long-term toxicity of AuNPs have not yet been investigated, and the high costs of production in large scale also remain a challenge [96].

#### 4.1.2. Nano-Scale Iron Oxides

Nano-scale iron oxides have exhibited great potential for biological applications [97]. Recently, a smart multifunctional nanostructure (SMN) containing an iron porous hollow magnetite NP (PHMNP), a heterobifunctional polyethylene glycol (PEG) ligand, and an aptamer sgc8 was constructed by Tan *et al.* [98]. In this case, multiple aptamers on the surface of a single SMN led to enhanced binding and uptake by target cancer cells due to the multivalent effect. The pores of the PHMNPs were stable at physiological pH, limiting the damage caused to healthy tissues by highly active anticancer agents. However, once the SMN reached the lysosomes of the target cancer cells through receptor-mediated endocytosis, the relatively low lysosomal pH levels resulted in the corrosion of the PHMNP pores, facilitating the release of DOX to kill the target cancer cells. The results showed that enhanced killing efficacy and improved targeting specificity could be achieved by using SMNs.

Among nano-scale iron oxides, superparamagnetic iron oxide NPs (SPIONs) have attracted significant attention as drug delivery platforms due to their special structure. SPIONs typically have one of the following two structural configurations: (i) a magnetic particle core is coated with a biocompatible polymer or (ii) a porous biocompatible polymer in which magnetic particles are precipitated inside the pores. The coating can be functionalized by attaching other molecules to increase the targeting yield. These molecules then act as attachment points for the coupling of cytotoxic drugs or target antibodies to the carrier complex [99,100]. Furthermore, SPIONs have several advantages, including stronger enhancement of proton relaxation and lower detection limits [101,102].

Ferumoxtran-10 (Combidex) is currently in phase III clinical trials for prostate cancer (PCa) imaging in which the dextran is coated with SPION [103]. The major shortcoming of Combidex is its inability to detect PCa disease outside the lymph nodes. To address this shortcoming, Farokhzad *et al.* [104] reported a novel thermally cross-linked SPION aptamer (TCL-SPION-Apt) bioconjugate in which the TCL-SPION was functionalized with an A10 RNA aptamer. DOX was intercalated in the aptamer and complexed with the TCL-SPION through charge interactions. The TCL-SPION-Apt bioconjugates could be used as therapeutic carriers for the selective delivery to PSMA-expressing cells. The cytotoxicity of Dox-loaded TCL-SPION–Apt bioconjugates was nearly as potent as free Dox. Using a similar process, aptamer-conjugated TCL-SPION was reported to be PCa-specific nanotheranostic agents [105]. These agents were capable for prostate tumor detection *in vivo* by simultaneous magnetic resonance imaging (MRI) and selective delivery of drugs to the tumor tissue. However, the exposure of the iron oxide core would cause toxicity in neurological systems; therefore, the potential of TCL-SPION-Apt bioconjugates as a targeted delivery system requires further validation prior to *in vivo* use. In addition, despite of the considerable potential benefits of SPIONs, SPIONs might still cause cellular disturbances, such as modulation of the actin cytoskeleton, alterations in gene expression profiles, and so on [106,107]. There is thus a need to identify potential cellular damages associated with these NPs.

Nano-scale iron oxides have attracted considerable attention as drug delivery platforms for their numerous advantageous properties, such as superparamagnetic property, low cytotoxicity, colloidal stability, and bioactive molecule conjugation capability. The cost of production is acceptable for application in drug delivery systems [97].

#### 4.1.3. Carbon Nanomaterials

Representative carbon nanomaterials that have been applied in aptamer-nanomaterial conjugated systems are single-walled carbon nanotubes (SWNTs). These materials are emerging as promising delivery vehicles for targeted drug delivery due to several important properties: (1) relatively easy internalization by cells; (2) a high capacity for drug loading and a large surface area for attaching specific targeting molecules either covalently or non-covalently; and (3) unique optical properties, such as NIR fluorescence and absorption, Raman scattering, and photoacoustic properties [108].

**Scheme 4:**
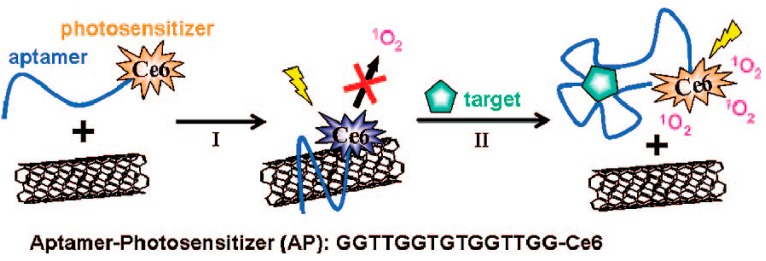
Schematic of aptamer-photosensitizer-SWNT complex and the regulation of SOG upon target binding. Reproduced with permission from Reference [109].

Tan *et al.* proposed a molecular complex AP-SWNT which was composed of a photosensitizer Ce6, an ssDNA aptamer (AP), and SWNTs for controllable singlet oxygen generation (SOG). Singlet oxygen is an alternative noninvasive cancer treatment generated during PDT [109]. The AP-SWNT design was based on the attachment of a DNA aptamer and photosensitizer (Ce6) to SWNTs, with the aptamer interacting noncovalently with the SWNTs by π-stacking between nucleotide bases and the SWNT side walls. When the target was absent, the SOG would be quenched by SWNT for the close proximity of the photosensitizer to the SWNT surface. However, in the presence of its target, the interaction between the aptamer and the target molecule could cause the DNA to fall off the SWNT surface, then lead to a restoration of SOG for PDT applications (Scheme 4). The resulting AP-SWNTs maintained the advantages of aptamers, presented an excellent specific response to thrombin and enhanced the efficiency and reliability of PDT. More specifically, when 2.0 μM thrombin was introduced, the singlet oxygen sensing of the AP-SWNTs exhibited a 13-fold enhancement.

Zhang’s group [110] constructed a multifunctional tumor-targeting drug delivery system, in which the SWNTs were used as drug carriers, AS1411 as a targeting ligand and DOX as a model chemotherapy drug. The delivery system could retain the optical properties of SWNTs and the cytotoxicity of DOX. More importantly, it could accumulate in tumors and facilitate the combination of chemotherapy and PTT, effectively promoting DOX cellular uptake and intracellular accumulation.

Graphene oxide is another carbon nanomaterial that has been applied in aptamer-nanomaterial conjugated systems. For example, Cai *et al.* [111] used cell type-specific aptamer-functionalized graphene oxide to deliver decitabine. In particular, the researchers conjugated graphene oxide to an aptamer A1 (a 45-base oligonucleotide that binds to A549 cell with high specificity and affinity) and then loaded decitabine onto the surface of the graphene oxide. The resulting complexes could specifically distinguish A549 cells from other types of cancer cells and subtypes of lung cancer cells. The drug release rate is much slower at physiological conditions than at acidic conditions. Importantly, cell viability assay results revealed that the complex displayed much higher therapeutic efficacy compared with the free drug.

Although these materials provide the advantages of low cost and high biocompatibility, the toxicological effects of carbon nanomaterials should also be considered. The concentration, physical form, degree of functionalization, and agglomeration state might all affect the toxicity of carbon nanomaterials [112,113].

#### 4.1.4. Mesoporous Silica Nanoparticles

Mesoporous silica nanoparticles (MSNs) are considered as the most promising candidate for targeted drug delivery because of the following unique properties: (1) a tunable particle size; (2) a stable and rigid framework; (3) a uniform and tunable pore size; (4) a high surface area and a large pore volume; (5) two functional surfaces; and (6) a unique porous structure [114].

Wang *et al.* [115] devised an efficient target drug delivery system for cancer therapy which was based on MSN–PEM–aptamer conjugate. In the system, MSNs were employed as drug carriers. Polyelectrolyte multilayers (PEMs) composed of thiolated poly(methacrylic acid) (PMASH) coated with the MSNs were used to prevent the premature leakage of drugs, and to controllably release drugs under reducing conditions. sgc8 was selected as the targeting recognition molecule. When this conjugate was exposed to a reducing environment, the deconstruction of PEM occurred for the cleavage of disulfide bonds and released the drug. The resulting drug delivery system combines the advantages of high drug-loading efficacy, stimulus responsiveness in the intracellular environment and high cell recognition ability.

Lu *et al.* [116] integrated a cancer-targeting DNA aptamer with mesoporous silica particles to construct a targeted drug delivery system. In this system, the nucleolin aptamer AS1411 was conjugated to the external MSN surface, and the drug molecules were encapsulated inside the nanopores. The functionalized MSN could selectively bind to nucleolin on the surface of MCF-7 breast cancer cells and was internalized via receptor-mediated endocytosis. This novel targeted drug delivery system showed pH-dependent controlled-release kinetics with specific binding to various target cells, thus ensuring the delivery of high doses of other therapeutics.

Similar to carbon nanomaterials, the targeted drug delivery systems based on MSNs is attractive for the numerous advantageous properties, such as low cost and high biocompatibility. However, many issues still need to be studied, such as the pharmacokinetics (PK) and pharmacodynamics (PD) of drugs loaded in MSNs, the acute and chronic toxicity, as well as the long-term *in vivo* degradation and compatibility of MSNs. In addition, the methods developed for synthesizing functional MSNs with a core-shell structure are limited to the production of a small amount of NPs [113,117].

#### 4.1.5. Quantum Dots

QDs (Quantum Dots) are microscopic metal or semiconductor boxes that hold a certain number of electrons. Due to their unique optical properties, including broad absorption with narrow photoluminescence spectra, a high quantum yield, low photobleaching, and resistance to chemical degradation, this material has been extensively investigated as a potential drug delivery vehicle [118,119].

**Scheme 5:**
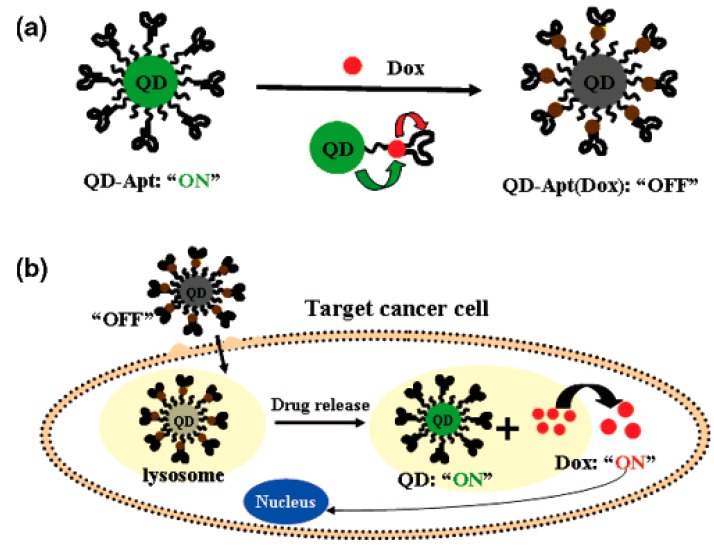
(**a**) Schematic illustration of QD-Apt(Dox) Bi-FRET system; (**b**) Schematic illustration of specific uptake of QD-Apt(Dox) conjugates into target cancer cell through PSMA mediate endocytosis. Reproduced with permission from Reference [120].

Farokhzad *et al.* [120] developed a novel QD-Apt-DOX conjugate. The conjugate was formed by the intercalation of DOX within the aptamer on the surface of QDs, and the QD and DOX fluorescence were quenched through a Bi-FRET mechanism (Scheme 5): the fluorescence of the QD was specifically quenched by DOX, while the fluorescence of DOX was simultaneously quenched by intercalation within the A10 PSMA aptamer. However, after selective uptake of the conjugate into the target cancer cell, the released DOX induced the recovery of fluorescence from both the QD and itself, thereby revealing the intracellular delivery of DOX and enabling the synchronous fluorescent localization and killing of cancer cells. The cytotoxicity of QD-Apt(Dox) conjugate was nearly equivalent potent to that of free Dox.

Savla *et al.* [121] reported the design and delivery of a tumor-targeted, pH-responsive QD-mucin1 aptamer-DOX (QD-MUC1-DOX) conjugate for chemotherapy for ovarian cancer. To achieve active cancer targeting, QDs were conjugated to a DNA aptamer specific for mutated MUC1, which is overexpressed in many cancer cells, including ovarian carcinoma. DOX was attached to the QDs via a pH-sensitive hydrazone bond to stabilize the complex in the systemic circulation and to allow drug release in the acidic environment inside cancer cells. The conjugate had higher cytotoxicity than free DOX in multidrug-resistant cancer cells and was preferentially accumulated in ovarian tumors *in vivo*.

Although QDs offer potentially invaluable societal benefits for targeted drug delivery, they may also pose risks to human health and the environment under certain conditions. In fact, several studies have strongly suggested that minor concentrations of QDs may be sufficient to cause long-lasting, even transgenerational, effects [122,123,124].

### 4.2. Organic Nanomaterials

#### 4.2.1. Liposomes

Due to their size and their dually hydrophobic and hydrophilic characteristics, liposomes are promising systems for drug delivery. Indeed, liposomes have been used successfully as drug delivery systems, and a number of liposome-based systems have been approved by FDA for disease treatment in the clinic [125,126]. Liposomes can both encapsulate hydrophilic therapeutic agents inside their aqueous core and load hydrophobic drugs within their lipid bilayer membranes. In certain approaches, the liposome surface can also be passivated with different ligands, such as PEG, extending liposomes’ systemic circulation time and resulting in preferential accumulation at tumor sites. Liposomes can be further improved by incorporating molecular recognition moieties, which can lead to drug transport with better efficacy and fewer side effects [127].

Lu *et al.* [128] developed aptamer-liposome bioconjugates that could effectively deliver cisplatin in a cancer cell-specific manner, and the selectivity of cisplatin was largely improved. The liposomes with an aptamer derived from AS1411 as a ligand, which had high binding affinity to nucleolin, could be internalized into cells through nucleolin-mediated endocytosis. The cell viability was approximately 59.5% at day 4 when the MCF-7 cells were treated with the aptamer-liposome-cisplatin, however, it was only 88.6% and 88.9% at day 4 for two control tests involving cisplatin. This study provided further evidence that the aptamer-mediated cancer-targeting strategy was highly specific and could be modulated for desired drug delivery applications.

Tan *et al.* [129] used a well-established liposome preparation protocol to prepare aptamer-modified liposomes. To increase liposome stability and to sustain aptamer stability in the serum, the liposomes were coated with the polymer PEG, and aptamers were conjugated to the surface of the liposomes to facilitate target binding. The formed sgc8 aptamer-liposome conjugates were able to selectively bind to their target cells, with no binding to non-target NB4 cells. In each liposome, there were approximately several thousand fluorescein isothiocyanate-dextran molecules, indicating a very high loading capacity.

Lu *et al.* [130] reported AS1411 aptamer-functionalized liposomes as a drug delivery system for targeted anticancer drug doxorubicin. The research showed that the liposomes could increase cellular internalization and cytotoxicity to MCF-7 breast cancer cells compared with non-targeted liposomes. Furthermore, improved antitumor efficacy against xenograft MCF-7 breast tumors in athymic nude mice was attributed to the liposomes’ enhanced tumor tissue penetration.

Kim *et al.* [131] designed novel anticancer drug-encapsulating liposomes which were conjugated to a PSMA-specific RNA aptamer. The resulting liposomes specifically bound to LNCaP prostate epithelial cells and, thus, significantly enhanced the *in vitro* cellular binding and uptake of the NPs compared with non-targeted NPs. Moreover, the liposomes were significantly more toxic to the targeted LNCaP cells than to non-targeted cancer cells. *In vivo* efficacy showed that the tumor was significantly reduced when was treated with the NPs as compared with the non-targeted NPs and free DOX.

Recently, Fattal *et al.* [132] successfully conjugated a 2ʹ-F-pyrimidine-containing RNA aptamer to the surface of PEGylated liposomes using the thiol-maleimide click reaction. The functionalized liposomes exhibited significantly enhanced cellular uptake compared with blank liposomes as well as promising potency as a specific drug delivery system.

#### 4.2.2. Poly(lactide-*co*-glycolic acid) Nanoparticles

Because poly(lactide-co-glycolic acid) (PLGA) is a biocompatible and biodegradable polymer that has been approved by FDA and has an established clinical safety record [133], drug delivery systems based on PLGA NPs play an important role in cancer therapy. PLGA NPs are usually functionalized with PEG because PEGylated polymeric NPs exhibit significantly reduced systemic clearance compared with similar particles without PEG [134,135].

**Scheme 6:**
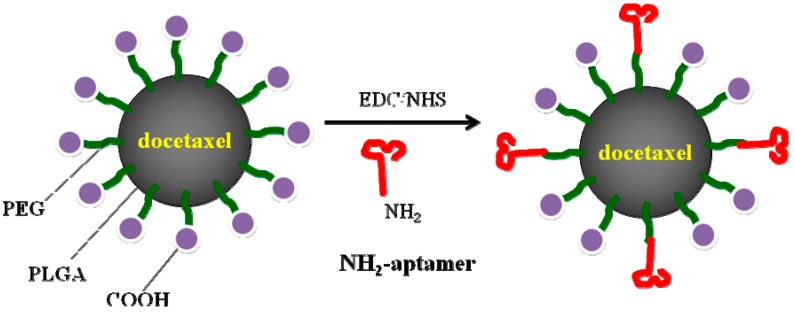
Schematic representation of the synthesis of PLGA-PEG-COOH copolymer and strategy of encapsulation of Dtxl. Adapted from Reference [136].

Farokhzad *et al.* [136] described docetaxel (Dtxl)-encapsulating NPs formulated with PLGA-*b*-PEG copolymer, wherein the surface was functionalized with A10 2ʹ-fluoropyrimidine RNA aptamers via an amide bond (Scheme 6). The resulting conjugates (Dtxl-NP-Apt) bound to the PSMA protein, which was expressed on the surface of LNCaP prostate epithelial cells, and were taken up by these cells, resulting in significantly enhanced *in vitro* cellular toxicity compared with non-targeted NPs lacking the PSMA aptamer. The Dtxl-NP-Apt conjugates also exhibited remarkable efficacy and reduced toxicity *in vivo*. Subsequently, A10 aptamer-functionalized PLGA-PEG NPs were used as a vehicle for transport of a platinum (Pt) (IV) prodrug. A Pt(IV) compound with alkyl chains at the axial positions was first prepared, after which the compound was encapsulated in PEGylated PLGA NP conjugates that bound to the A10 2′-fluoropyrimidine RNA aptamer. These resulting aptamer-derivatized Pt(IV)-encapsulating NPs were significantly superior to cisplatin or non-targeted NPs in terms of activity against LNCaP cells [137]. The conjugate was then investigated *in vivo*, and the results showed that the 10-days maximum tolerated doses following a single intravenous injection of the conjugate in rats and mice were 40 and 5 mg/kg, respectively. PK studies with the conjugate revealed prolonged Pt persistence in the systemic blood circulation and decreased accumulation of Pt in the kidney. Moreover, the conjugate exhibited significant dose-sparing characteristics with regard to the drug, with equal efficacy at 1/3 the dose of cisplatin administered in its conventional form [138].

Farokhzad *et al.* [139] prepared a biointegrated triblock copolymer (TCP) consisting of PLGA, PEG, and the A10 aptamer (Apt). This TCP could be used for the self-assembly of targeted NPs for PCa targeting. The researchers found that increasing the Apt density on the NP surface could increase the rate of NP uptake by LNCaP cells *in vitro* and that a high Apt surface density could increase NP accumulation in the liver and spleen. Therefore, it was necessary to balance the tumor-targeting ligand surface density and the antibiofouling surface properties. Using different ratios of functional biomaterials (PLGA-PEG conjugated to aptamer) to non-functional biomaterials, the surface aptamer density could be adjusted, and the optimal ratio for maximal PCa cell uptake both *in vitro* and *in vivo* was identified.

Tan *et al.* [140] synthesized aptamer-coated PLGA hybrid NPs with core-shell lipid-polymeric structures via simple nanoprecipitation and self-assembly. After self-assembly, hydrophobic PLGA with encapsulated hydrophobic paclitaxel (PTX) constituted the core structure, and lecithin, DSPE-PEG and lipid-PEG-aptamer loaded with DOX formed the hydrophilic shell. The cytotoxicity results showed that this targeted co-delivery system selectively enhanced antitumor efficacy.

#### 4.2.3. Polymeric Micelles

Polymeric micelles (PMs) can be used for drug targeting due to their inherent properties, including stability in plasma and longevity *in vivo*. Additionally, the pathological characteristics of tumors allow PMs to be targeted to the tumor site via enhanced permeability and retention. The amphiphilic block copolymer of PMs is particularly suitable for the encapsulation of hydrophobic drugs, and PMs can be conjugated to many ligands on their outer shell to bring the micelles to cancer cells [141] The aptamer-micelles can simply interact with the cell membrane and quickly release the doped hydrophobic molecules into the cells [142].

**Scheme 7:**
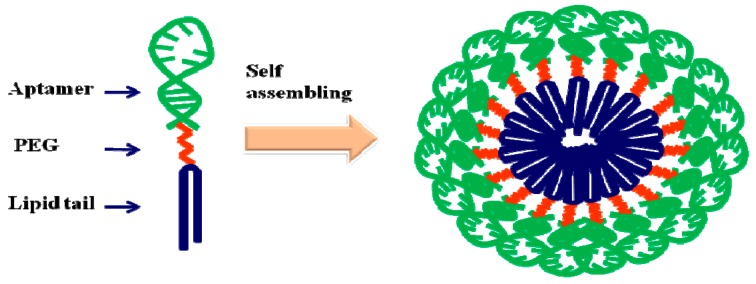
Schematic illustration of aptamer–micelle formation. Adapted from Reference [142].

Tan *et al.* [142] reported the design of a self-assembled aptamer-micelle nanostructure (Scheme 7). In the aptamer assembly, the aptamer TDO5 not only acted as the building block for the nanostructure but also performed a recognition function, identifying its specific target. Interestingly, this nanostructure showed an 80-fold enhancement in selective binding to Ramos cells at 37 °C compared with the free aptamer. Furthermore, the aptamer-micelles showed great dynamic specificity in flow channel systems used to mimic drug delivery in a flowing system.

Hao *et al.* [143] developed novel aptamer-functionalized PEG-polylactic acid micelles (APPs) with the objective of targeting the transferrin receptor on brain endothelial cells. Flurbiprofen was loaded into the APPs using the co-solvent evaporation method. The experimental results indicated that the APPs significantly enhanced the surface association of micelles with bEND5 cells. Furthermore, the APPs significantly enhanced intracellular flurbiprofen delivery compared with unmodified micelles.

Gong *et al.* [144] prepared aptamer-conjugated unimolecular micelles that were formed by individual hyperbranched polymer molecules, consisting of a hyperbranched H40 polymer core and approximately 25 amphiphilic polylactide (PLA)-PEG block copolymer arms (H40-PLA-PEG-Apt). The micelles exhibited pH-sensitive, controlled drug release and much higher cellular uptake among PSMA-positive CWR22Rn1 prostate carcinoma cells than non-targeted unimolecular micelles. In CWR22Rn1 tumor-bearing mice, DOX-loaded H40-PLA-PEG-Apt micelles exhibited much higher levels of DOX accumulation in the tumor tissue than DOX-loaded H40-PLA-PEG micelles.

Qiu *et al.* [145] developed an AS1411-functionalized composite micelle (CM-Ap) composed of AS1411 (Ap)-modified Pluronic F127, β-cyclodextrin-linked PEG-b-PLA and DOX. The study revealed that the cellular uptake of CM-Ap was higher than the uptake of untargeted CM due to the nucleolin-mediated endocytosis effect. *In vivo* testing also demonstrated that the CM-Ap exhibited a prolonged circulation time in the blood, enhanced accumulation in tumors, improved antitumor activity, and decreased cardiotoxicity.

Chen *et al.* [146] developed Apt-mixed micelles consisting of a pH-responsive copolymer, d-α-tocopherol PEG 1000-block-poly-(β-amino ester) (TPGS-b-PBAE, TP), and AS1411 aptamer (Apt)-decorated TPGS polymer (Apt-TPGS). PTX was encapsulated in the Apt-mixed micelles. The resulting PTX/Apt-mixed micelles were stable at pH 7 but dissolved and quickly released the encapsulated PTX in a weakly acidic environment (pH 5.5). Compared with non-Apt-modified mixed micelles, more Apt-modified mixed micelles were internalized by SKOV3 ovarian cancer cells, whereas no remarkable difference in cellular uptake was observed in normal cells (LO2 cells). Moreover, the micelles exhibited significantly increased cytotoxicity and G2/M phase arrest among SKOV3 cells compared with free PTX.

Although the past decade has witnessed the explosive development of biodegradable micelles for targeted and controlled anticancer drug delivery, certain challenges are yet to be addressed. In particular, the techniques are still far from optimal due to issues such as low *in vivo* stability, poor tumor penetration, inefficient cellular uptake, and slow intracellular drug release [147].

#### 4.2.4. Dendrimers

Dendrimers have narrow polydispersity and a nanometer size range, allowing easier passage across biological barriers compared with other architectural forms of polymers that have been used in drug delivery systems [148,149]. Combination with the target specificity and sensitivity of an aptamer has resulted in aptamer-dendrimer bioconjugates, which have opened new vistas for targeted drug delivery [150]. Dendrimers are hyperbranched polymers characterized by a central inner core surrounded by layers of repeating units, with an outermost layer of multivalent functional groups. The cavities inside the dendritic structure can be modified to incorporate hydrophobic and hydrophilic drugs, and the outermost layer of functional groups can be modified to attach aptamers for tumor recognition [151].

Jon *et al.* [152] used poly(amidoamine) (PAMAM) succinamic acid dendrimers as vehicles for targeted drug delivery. Firstly, an oligodeoxynucleotide was conjugated to PAMAM dendrimers, resulting in a single-stranded ONT-conjugated dendrimer (sONT-DEN). Secondly, an A9 RNA aptamer was hybridized with the sONT-DEN to create a double-stranded Apt-dONT-DEN conjugate. Finally, DOX was intercalated into the double-stranded dONT site to yield DOX-loaded Apt-dONT-DEN conjugates. The resulting conjugates showed excellent antitumor efficacy and target specificity in an *in vivo* prostate tumor model. The conjugates also exhibited high drug-loading capacity and enhanced the *in vivo* stability of the oligonucleotides.

However, dendrimers also have limitations, including the complex and costly multistep procedures involved in the synthesis and processing of dendrimer-based NPs. Future work will be necessary to identify cost-effective synthesis strategies and to determine the relationship between dendrimers and drug molecules for successful commercialization of this technology [149].

#### 4.2.5. Serum Albumin Nanoparticles

Serum albumin NPs have been extensively studied for drug delivery because they are biodegradable, relatively easy to prepare and have a controllable size range [153]. The hydrophobic albumin core of these NPs displays a high affinity for hydrophobic drugs, and the resulting combinations may form exceptional slow-release formulations under physiological conditions. As the primary structure of proteins contains –NH_2_, –COOH, –SH and other functional groups, serum albumin NPs can offer various possibilities for conjugating target molecules to particulate surfaces [154].

Gao *et al.* [155] prepared bovine serum albumin (BSA) NPs using a desolvation technique and coated them with PAH/PSS multilayers to activate their surface, to which the AS1411 aptamer was covalently bound. The functional BSA NPs were then loaded with DOX. The resulting conjugates exhibited a faster release rate at pH 5.5 than at pH 7.4. With this pH-responsive release and targeting properties, the BSA NPs loaded with DOX could induce the death of liver cancer cells more effectively than the free drug could while maintaining the same low toxicity to normal liver cells.

#### 4.2.6. DNA Nanomaterials

Although various nanostructures have been applied in targeted drug delivery, there is still demand for a simple, target-specific and economical drug delivery platform with high maximum tolerated doses. Therefore, a series of DNA nanomaterials have been proposed as drug delivery platforms by Tan *et al.* [156,157,158,159,160].

**Scheme 8:**
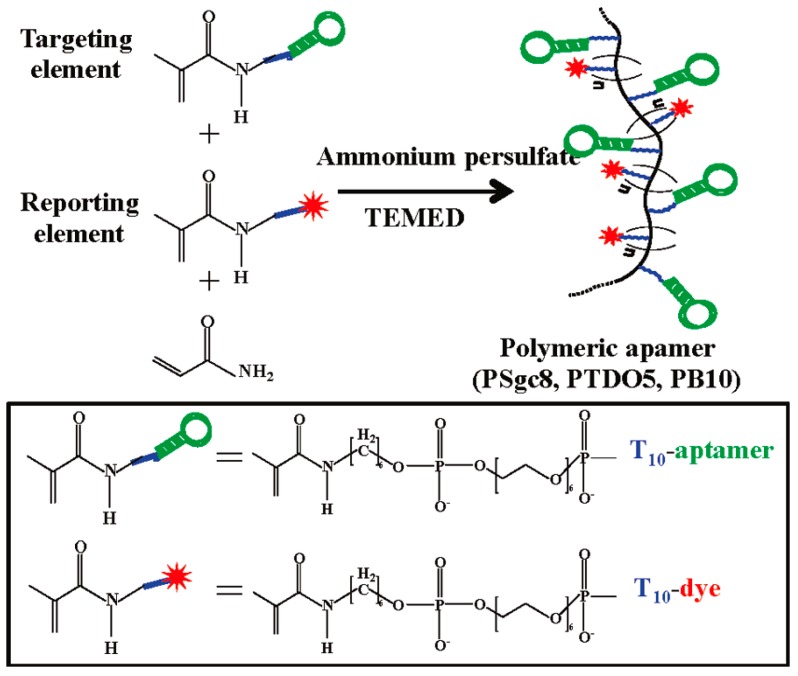
Schematic of polymeric aptamer synthesis. Reproduced with permission from Reference [156].

Tan *et al.* [156] firstly reported targeted drug delivery employing polymeric aptamers to induce selective cytotoxicity inside target cells. Conjugates were specifically assembled by polymerization using a one-step procedure (Scheme 8). A reporting element, or 5′-acrydite-T_10_-dye-3′, was introduced to maintain the appropriate configuration of the individual aptamers and to provide a tracking signal for both targeting and internalization. The targeting elements, or 5′-acrydite-aptamers, on the polymer chain could facilitate cellular delivery by multivalent binding. The resulting polymeric conjugate exhibited target recognition capability, enhanced cellular internalization, and cellular disruption. Importantly, the polymer backbone built into the conjugate was cytotoxic only inside cells. As a result, selective cytotoxicity was achieved equally in both normal cancer cells and drug-resistant cells.

**Scheme 9:**
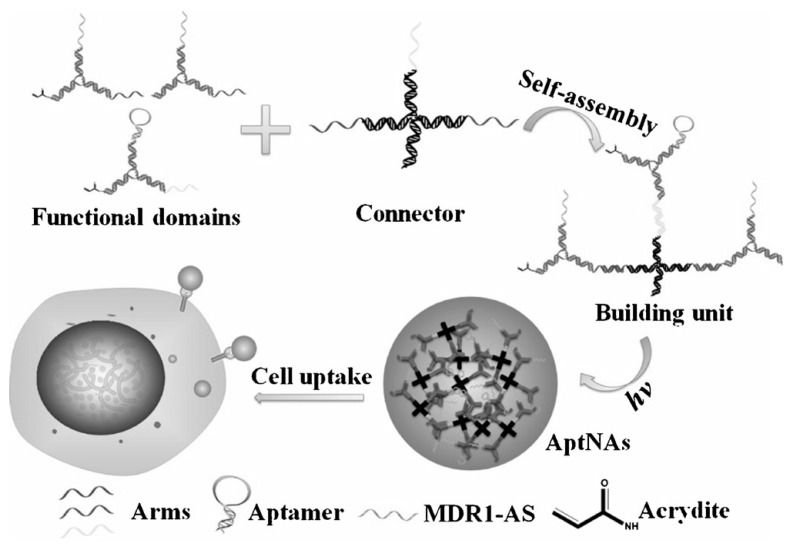
Schematic illustration of the multifunctional self-assembled nanoassembly building units and photo-cross-linked nanoassembly structure. Reproduced with permission from Reference [157].

As another example, Tan *et al.* [157] constructed a multifunctional aptamer-based DNA nanoassembly (AptNA) for targeted cancer therapy. The researchers first used base-pair hybridization to design the multifunctional AptNA with various Y-shaped functional DNA domains, including targeting aptamers, intercalated anticancer drugs, and therapeutic antisense oligonucleotides. These functional DNA domains were then linked to an X-shaped DNA core connector, termed a building unit. Finally, hundreds of these basic building units were further photo-cross-linked into a multifunctional aptamer-based nanoassembly structure (Scheme 9). The resulting AptNAs demonstrated excellent biostability in a physiological environment (pH 7.4), thus avoiding unnecessary leakage of intercalated drugs during delivery.

Recently, Tan *et al.* [158,159] presented monodisperse multifunctional DNA nanostructures assembled through DNA liquid crystallization, termed nanoflowers (NFs). These NFs were integrated with aptamers, bioimaging agents, and drug-loading sites. Compared with conventional DNA nanostructure assembly, NF assembly was independent of template sequences, avoiding the otherwise complicated design of DNA building blocks assembled into nanostructures by base pairing. The resulting NFs exhibited high fluorescence intensity and excellent photostability. Moreover, these NFs were demonstrated to exhibit selective cancer cell recognition and targeted anticancer drug delivery and to be of use in bioimaging.

Additionally, Tan *et al.* [160] designed and developed smart self-assembled, aptamer-tethered DNA nanotrains (aptNTrs) for the targeted transport of molecular drugs as cancer theranostics. In particular, long aptNTrs were self-assembled from only two short DNAs upon initiation by modified aptamers and worked similarly to locomotives, guiding nanotrains toward target cancer cells. Meanwhile, tandem “boxcars” served as carriers with high payload capacity, transporting drugs to target cells and inducing selective cytotoxicity. The aptNTrs enhanced the maximum tolerated dose in non-target cells, and the potent antitumor efficacy and reduced side effects of drugs delivered by biocompatible aptNTrs were demonstrated in a mouse xenograft tumor model.

Except the cytotoxic agent and photosensitizer, there was also a series of attempts to the delivery of siRNA. For example, Rossi *et al.* [161] used the novel dual inhibitory function aptamer-siRNA delivery system for HIV-1 therapy. Haam *et al*. [162] used the aptamer conjugated polyplexes (APs) to investigate the synergism between shRNAs against Bcl-xL and doxorubicin (DOX) in combination cancer therapy. Yu *et al.* [163] used CTLA4 aptamer delivering STAT3 siRNA to tumor-associated and malignant T cells.

In summary, nanomaterials possess many advantages over bulk structures. Due to the high selectivity and affinity of aptamers, aptamer-nanomaterial conjugates are ideal agents for drug delivery, with proven therapeutic effects and reduced toxicity to normal tissue. However, the unforeseen hazardous properties of the carrier nanomaterials themselves should be considered, and drug delivery systems based on biocompatible and biodegradable nanomaterials should be promising for either FDA approval or clinical trial.

## 5. Conclusions

Since their discovery in the 1990s, aptamers, with their high affinity and specific targeting abilities, have been widely used in targeted drug delivery systems. However, the conventional aptamer SELEX selection procedure is time consuming and labor intensive. Further refinements of CELL-SELEX techniques are needed to facilitate the identification of more disease targets and to improve targeting efficiency.

Due to the wide diversity of potential targets, no standard SELEX methods have been established so far, although different methods have been used for aptamer screening. After a decade of development, both the screening efficiency and applications have been improved. The targets have changed from single substances to animal cells and pathogens (bacteria). At the same time, complex screening systems such as multi-target screening and automated screening have emerged. In order to improve their efficiency, new outcomes from modern biophysical chemistry research and bio-separation technologies need to be integrated into SELEX so that high-throughput screening technologies can be developed. SELEX technologies will significantly push forward the studies related to gene regulation, proteomics, biochemical analysis, disease treatments and new drug exploration.

Furthermore, because aptamers are nucleic acids, they are highly susceptible to degradation by several nucleases *in vivo*, and the small size of aptamers also makes them liable to renal filtration. Consequently, aptamers are often modified with different types of agents, resulting in increased cost and side effects. Therefore, methods to reduce the cost of aptamer SELEX selection will be needed in the future.

Chemically conjugated targeted drugs exhibit good chemical stability and isotropic properties for targeted delivery to tumor cells (Table 2). However, low drug loading and the complex and costly multistep procedures involved in the design and synthesis have retarded the development of this approach. At the same time, the desirable properties of nanomaterials make them good candidate signal generation and transduction components as well as delivery vehicles. Compared with chemical conjugated targeted drugs, the aptamer-nanomaterial conjugated systems showed large drug loading. However, complex and costly preparation procedures, the PK and toxicology of nanomaterials require further investigation and development, and there are great difficulties in ascertaining the risks associated with this new technology. Chemically conjugated targeted drugs based on DNA nanotrains exhibit not only good chemical stability and isotropic properties but also a high loading capacity, biocompatibility and biodegradability, and may be a good choice for targeted drug delivery [52].

**Table 2:** The advantages and disadvantages of the aptamer-functionalized targeted drug delivery systems.

Aptamer-Functionalized Targeted Drug Delivery Systems	Advantage	Disadvantage
Aptamer-small molecule conjugated systems	Good chemical stability, isotropic properties	Low drug loading, complex and costly procedures
Aptamer-nanomaterial conjugated systems	High loading capacity	Unpredictable risks, complex and costly procedures

In summary, aptamer-based delivery systems appear to have potential for use in cancer therapy. However, major challenges and issues are yet to be resolved. Therefore, the study and development of promising therapeutic strategies is ongoing.
